# Social trust, interpersonal trust and self-rated health in China: a multi-level study

**DOI:** 10.1186/s12939-016-0469-7

**Published:** 2016-11-08

**Authors:** Zhixin Feng, Athina Vlachantoni, Xiaoting Liu, Kelvyn Jones

**Affiliations:** 1 Centre for Research on Ageing, Social Sciences, Faculty of Social, Human and Mathematical Sciences, University of Southampton, Southampton, UK; 2 Centre for Research on Ageing and ESRC Centre for Population Change, Social Sciences, Faculty of Social, Human and Mathematical Sciences, University of Southampton, Southampton, UK; 3 School of Public Affairs, Zhejiang University, 866 Yuhangtang Road, Hangzhou, Zhejiang Province 310058 China; 4 School of Geographical Sciences, University of Bristol, Bristol, UK

**Keywords:** China, “in-group” trust, “out-group” trust, Self-rated health, Multi-level modelling

## Abstract

**Background:**

Trust is important for health at both the individual and societal level. Previous research using Western concepts of trust has shown that a high level of trust in society can positively affect individuals’ health; however, it has been found that the concepts and culture of trust in China are different from those in Western countries and research on the relationship between trust and health in China is scarce.

**Method:**

The analyses use data from the national scale China General Social Survey (CGSS) on adults aged above 18 in 2005 and 2010. Two concepts of trust (“out-group” and “in-group” trust) are used to examine the relationship between trust and self-rated health in China. Multilevel logistical models are applied, examining the trust at the individual and societal level on individuals’ self-rated health.

**Results:**

In terms of interpersonal trust, both “out-group” and “in-group” trust are positively associated with good health in 2005 and 2010. At the societal level, the relationships between the two concepts of trust and health are different. In 2005, higher “out-group” social trust (derived from trust in strangers) is positively associated with better health; however, higher “in-group” social trust (derived from trust in most people) is negatively associated with good health in 2010. The cross-level interactions show that lower educated individuals (no education or only primary level), rural residents and those on lower incomes are the most affected groups in societies with higher “out-group” social trust; whereas people with lower levels of educational attainment, a lower income, and those who think that most people can be trusted are the most affected groups in societies with higher “in-group” social trust.

**Conclusion:**

High levels of interpersonal trust are of benefit to health. Higher “out-group” social trust is positively associated with better health; while higher “in-group” social trust is negatively associated with good health. Individuals with different levels of educational attainment are affected by trust differently.

## Background

Trust is important for health at both the individual and societal level, and it is an important component for the smooth functioning of society, as it contributes to the development, maintenance and sustainability of the social quality of people’s lives [29]. As a core aspect of social capital, trust has been found to exert protective effects for health [17]. A growing body of studies have shown that higher levels of trust are associated with better self-rated health [8], lower mortality [16], and better wellbeing [30].^1^ Moreover, lower levels of trust are associated with higher rates of most major causes of death, including heart disease, cancers, and violent deaths [24],and have preceded a change from good health to poor health [5], along with a decline in happiness [26]. The mechanisms to explain the protective effects of higher levels of trust on population health vary [12], pointing to higher levels of trust promoting more rapid diffusion and uptake of health-promoting innovations; increasing the likelihood of healthy norms of behaviour being adopted; and exerting social control over deviant health-related behaviour.

In terms of the definition of trust, Taormina [25], a social psychologist, defined trust as “a conviction that another person will perform certain actions, or behave as promised”; and he pointed out that there are cultural differences in the concepts of trust between Western countries and China. In Western countries, individualism is embedded in the culture, with every individual displaying a different degree of trust towards other people; while, the Chinese culture points to a strong concept of collectivism and Chinese people have unconditional trust in their own family (the core “in-group”), but distrust anyone else who is not in one’s family and is thus a member of the “out-group” [25, 28]. This is due to the moral behaviour (and by extension trust) emanating from the Confucian social ethics, which is closely linked to concrete personal connections, and lacks “rules for impersonal dealings beyond the face-to-face level” [23]. Thus, complex networks exist across Chinese societies, and are primarily based upon particularized trust (“in-group” members), which remains limited to close and concrete relationships. All other individuals who are not family members have to prove that they can be trusted by being consistently honest and reliable over a long-term time frame, in order to become members of one’s “in-group” [25]. Therefore, Chinese society can be characterized as “high-trust” society as Chinese people show a high level of trust towards members of their own “in-group”. At the same time, Chinese society also can be characterized as a “low-trust” society as Chinese people tend to have a “pervasive distrust of strangers” (“out-group” members) ([3], pp. 69-95).

On the other hand, the concepts of measurements of trust are also different between Western countries and China [27]. The globally standard question “generally speaking, would you say that most people can be trusted or that you can’t be too careful in dealing with people” is the standard measure of social trust which is assumed to capture the underlying meaning of “trust in strangers” [27]. Torpe and Lolle [27] compared different measurements of social trust (i.e. trust most people, trust strangers, trust people of another religion, and trust people of another nationality) across the world, and they found that the variable indicating whether “most people can be trusted” has been considered as an indicator of “trust in strangers” in Western countries; while, in China, they found that the majority of Chinese respondents generally trust most people but do not trust strangers, or people with a different nationality or religion. This may imply that Chinese individuals may understand “most people” as individuals they already know (members of one’s “in-group”). Given this, Torpe and Lolle [27] questioned the validity of previous studies of social trust based on the standard question of whether “most can be trusted”, while Taormina [25] suggested that a solution to the culturally-specific distinction between “in-group” and “out-group” trust for Chinese respondents is to ask a question referring to more salient persons (i.e. only “out-group” members).

Most research linking trust to health has, to date, been conducted in developed, Western societies, while such research in China is scarce. In addition, although there are two published studies which find a positive effect of trust on health in China (i.e. [15, 30]), these studies integrated questions of trust relating both to individuals that the respondent already knew (i.e. members of one’s “in-group” such as neighbours, relatives or colleagues) and to strangers (members of one’s “out-group”), which does not pay attention to the unique trust environment (higher “in-group” trust than “out-group” trust) in Chinese society. The effects of such an “outlier” definition of social trust in China on health may be different from Western countries, and to the best of our knowledge, no literature has discussed these issues in China. This study will explore the relationship between the two concepts of trust (namely “out-group” and “in-group” trust) and the report of health status in China.

## Data and methods

### Data

Data were collated from the China General Social Survey (CGSS) conducted by the Department of Sociology of Renmin University of China and the Social Science Division of the Hong Kong Science and Technology University. The survey has been a regular cross-sectional survey of urban and rural Chinese households since 2003. Households and eligible household members are randomly selected to represent each sample household in mainland China. To date, five surveys have been conducted in 2003, 2005, 2006, 2008 and 2010. Only those surveys conducted in 2005 and 2010 contain information on trust and health and are therefore used in this study.^2^ The final sample for the data was collected from 9,458 and 10,100 individuals aged 18 and above, along with the complete data from the 2005 and 2010 surveys respectively. This data has a two-level natural hierarchical structure with individuals nested within provinces.

Self-Rated Health (SRH) is the dependent variable in this paper. Although SRH is a subjective measure of health, many studies find that it is a sensitive and reliable indicator of current and future health status [11]. Respondents were asked “How would you rate your health in the last month?”, rating their health according to the following categories: excellent, very good, good, fair, poor or very poor (for the 2010 dataset, SRH categorised the levels as: very good, good, fair, poor or very poor). For comparability with previous studies (i.e. [8, 15, 24]), we reclassified the categories to form a dichotomous outcome of SRH in which 1 represented Good health (excellent, very good or good) and 0 represented Other categories (fair, poor or very poor). The distributions of SRH for the two years are presented in Table 1. Approximately 61.7 % and 58.1 % of the sample population reported good health in 2005 and 2010 respectively, and there are no substantial differences in the distributions of SRH between the two years.

**Table 1:** Descriptive statistics for individual and province level variables

	2005 “Out-group” trust	2010 “In-group” trust
Response		
Self-rated health	Excellent, very good or good (61.7 %), Fair, poor or very poor health (38.3 %)	Very good or good (58.1 %), Fair, poor or very poor health (41.9 %)
Predictors		
*Level 1*	*Individuals, n = 9,458*	*Individuals, n = 10,100*
Age	18-94, mean = 44	18-96, mean = 47
Gender	Male (48 %), Female (52 %)	Male (49.8 %), Female (50.2 %)
Education attainment	None (10.4 %), Primary (24.7 %), Junior high (30.9 %), Senior high (22.5 %), above high (11.5 %)	None (12.7 %), Primary (23.7 %), Junior high (29.9 %), Senior high (19.1%), above high (14.6 %)
Residence	Urban (58.4 %); Rural (41.6 %)	Urban (60 %); Rural (40 %)
Personal annual income (unit:10,000 Yuan)	0-40, mean = 0.88	0-600, mean = 1.92
Interpersonal trust	Low trust (75.5 %), Average trust (19.2 %), High trust (5.3 %)	Low trust (23.9 %), Average trust (9.8 %), High trust (66.3 %)
*Level 2*	*Provinces, n = 28*	*Provinces, n = 31*
Aggregated social trust	8.7 % ~ 52.7 %, mean = 25.9 %	63 % ~ 85.7 %, mean = 75 %

In terms of the independent variables, trust is the key variable of interest. Usefully, the measurements of trust are different in 2005 and 2010, thereby capturing two different concepts of trust. In 2005, perceptions of interpersonal trust were collected through individual responses to the question “Generally speaking, without a direct pecuniary interest, do you trust strangers?” with the potential responses being “The majority/most people can’t be trusted” defined as low trust, “About half of the people can be trusted, while the other half cannot be trusted” defined as average trust and “The majority/most people can be trusted” defined as high trust. This question directly measured the “out-group” interpersonal trust according to Taormina’s [25] suggestion which is to refer to more salient persons (i.e. strangers). In 2010, the questionnaire used a standard trust question of “Generally speaking, do you agree most people can be trusted?”, and individuals responded through the following categories: “Completely disagree/disagree” which indicated low trust, “Approximately half of the people can be trusted while the other half cannot be trusted” referring to average trust and “Completely agree/agree” indicating high trust. In the preliminarily analysis of this 2010 dataset, we found that a high proportion of Chinese respondents who trust most people, also trust their family member(s), relative, friends, colleagues and people from the same hometown. This is consistent with Torpe and Lolle’s [27] findings which may imply that Chinese individuals consider “most people” to mean people that they already know. Therefore, we treat this variable as an indicator of “in-group” interpersonal trust. Building on existing literature which has taken other demographic and socio-economic factors into account, we also consider age, gender, education attainment, urban-rural residence, and total personal annual income (including waged income, business income, property income, and other income) as “control” variables. The summary statistics for these variables are given in Table 1. In terms of the “out-group” and “in-group” trust at the societal level, we aggregated a social trust variable at the province level (a continuous scale) from the percentage of respondents showing average or high trust from their individual responses. The values of aggregated social trust were calculated by taking the arithmetic average of the weighted individual responses. As Table 1 shows, the Chinese society was a low “out-group” trust society in 2005 and a high “in-group” trust society in 2010. These two concepts of trust will allow us to explore the effect of different concepts of trust on individuals’ health in China.

## Methods

Since the response of Self-Rated Health is binary (Good health versus other categories of health status) and the data has a hierarchical structure, a multi-level logistical model based on a logit-link function was used. Multi-level statistical techniques provide an analytical framework for data with a hierarchical structure [2]. These techniques can analyse the effects of individual characteristics (interpersonal trust) and province characteristics (social trust variable) on health simultaneously [7, 9].

A simple multi-level logistic model is shown below as:

$$ loge\;\left({Y}_{ij}\right)={\upbeta}_0+{\upbeta}_1{\mathrm{X}}_{1\mathrm{i}\mathrm{j}}+{\upbeta}_2{\mathrm{X}}_{2\mathrm{j}}+{u}_{0j} $$

1 $$ \left[{u}_{0j}\right]\sim \mathrm{N}\;\left(0,\;{\upsigma}_{u0}^2\right) $$

Where *Y*_*ij*_ is 1 if the individual i in province j reporting excellent, very good or good health; and 0 if they report other health statuses. X_1ij_ is an individual characteristic predictor (i.e. age, gender, education, income and personal trust) at the individual level, and X_2j_ is a social trust variable at the province level. The βs are the “fixed” or average values across all provinces so that the β_1_ parameter captures the “micro” individual characteristics effect, and the β_2_ parameter captures the “macro” social trust effect at the province level. The u_0j_ terms are the random differential intercepts which represent province-level residual differences after taking account of individual characteristics and social trust; these are shown on the logit scale and are assumed to be normally distributed with a mean of 0 and variance of σ_*u*0_^2^. This variance term summarizes the residual (after taking account of included variables) between province variations on the logit scale.

All the models were estimated using the *MLwiN* 2.30 software [19]. Due to the discrete nature of the outcome and because there are 28 higher level units, Bayesian MCMC estimations were applied for more robust estimation [1, 10]. This form of estimation also allows for the calculation of the Deviance Information Criterion (DIC) which provides a comparative measure of goodness-of-fit between models. The smaller the DIC value is, the better the model is [22].

## Results

Table 2 shows the results of the multi-level logit models for “out-group” trust and health in 2005. The models are increasingly more complex using the DIC comparison. Models 1 to 3B are established from the null model, where individuals (level 1) are nested within provinces (level 2) with no predictor variables, shifting to a model including age (in a linear form), gender, educational attainment, urban-rural residence, personal income (a quadratic term), interpersonal trust (“out-group” trust at the individual level) and social trust (“out-group” trust at the province level).

**Table 2:** Logit multi-level regression estimates for 2005 (“Out-group” trust)

	Model 1	Model 2	Model 3A	Model 3B	Model 4A_2005	Model 4B_2005	Model 4C_2005
	Estimate (S.E.)	Estimate (S.E.)	Estimate (S.E.)	Estimate (S.E.)	Estimate (S.E.)	Estimate (S.E.)	Estimate (S.E.)
Fixed Part							
CONS	0.459 (0.075)***	0.042 (0.118)	-0.003 (0.109)	0.029 (0.103)	0.036 (0.101)	0.024 (0.114)	0.032 (0.106)
Age		-0.045(0.002)***	-0.045 (0.002)***	-0.045 (0.002)***	-0.045 (0.002)***	-0.045 (0.002)***	-0.045 (0.002)***
Male (ref: Female)		0.299 (0.049)***	0.296 (0.048)***	0.293 (0.049)***	0.290 (0.048)***	0.294 (0.048)***	0.295 (0.049)***
Educational attainment (ref: None)							
Primary		0.051 (0.084)	0.046 (0.084)	0.038 (0.084)	0.036 (0.085)	0.037 (0.088)	0.037 (0.081)
Junior		0.231 (0.089)**	0.229 (0.089)***	0.219 (0.089)**	0.212 (0.089)**	0.222 (0.094)**	0.218 (0.084)**
Senior		0.277 (0.098)***	0.265 (0.099)***	0.254 (0.098)**	0.250 (0.100)**	0.253 (0.104)***	0.254 (0.097)**
Above		0.202 (0.120)*	0.196 (0.121)*	0.183 (0.122)	0.181 (0.121)	0.180 (0.125)	0.183 (0.118)
Rural (ref: urban)		0.225 (0.058)***	0.216 (0.060)***	0.213 (0.058)***	0.216 (0.058)***	0.223 (0.058)***	0.213 (0.059)***
Log personal income		0.206 (0.031)***	0.207 (0.031)***	0.21 (0.030)***	0.209 (0.030)***	0.211 (0.030)***	0.206 (0.031)***
Log personal income^2^		0.062 (0.018)***	0.059 (0.018)***	0.059 (0.018)***	0.058 (0.018)**	0.059 (0.018)***	0.058 (0.018)***
Interpersonal trust (ref: Low trust)							
Average			0.241 (0.062)***	0.23 (0.060)***	0.228 (0.061)***	0.225 (0.061)***	0.227 (0.061)***
High trust			0.389 (0.110)***	0.378 (0.110)***	0.375 (0.110)***	0.370 (0.107)***	0.374 (0.110)***
Social trust				0.016 (0.006)**	0.027 (0.009)***	0.010 (0.007)**	0.015 (0.006)
*Interactions:*							
Social trust * Education (ref: social trust * None)							
Social trust * Primary					0.001 (0.009)		
Social trust * Junior					-0.018 (0.008)**		
Social trust * Senior					-0.019 (0.009)**		
Social trust * Above					-0.014 (0.011)		
Social trust * Rural						0.014 (0.006)**	
Social trust * Log personal income							-0.006 (0.003)**
Random Part							
Level 2							
Between Province	0.146 (0.047)***	0.129 (0.043)***	0.123 (0.041)***	0.095 (0.034)***	0.101 (0.036)***	0.105 (0.038)***	0.101 (0.036)***

***p < 0.01, **p < 0.05, *p < 0.1

^2^ stands for the Square form of log personal income in the model

The results of Model 3B show that higher percentages of “out-group” social trust (at the province level) are associated with higher odds of reporting good health (ORs = 1.02, 95 % CI: 1.00-1.03); for the “out-group” interpersonal trust (individual level), people who trusted strangers are 45% more likely to report good health than those who did not trust strangers (95 % CI: 1.18-1.80). Even those who reported average trust are 26 % more likely to report good health than those who did not trust strangers at all (95 % CI: 1.12-1.42). For other control variables, older people, women, those with no formal educational attainment, those who live in urban areas and low income groups in general report worse health than younger people, men, those with higher educational attainments (junior or senior level), rural residents and those on higher incomes, respectively.

**Fig. 1:**
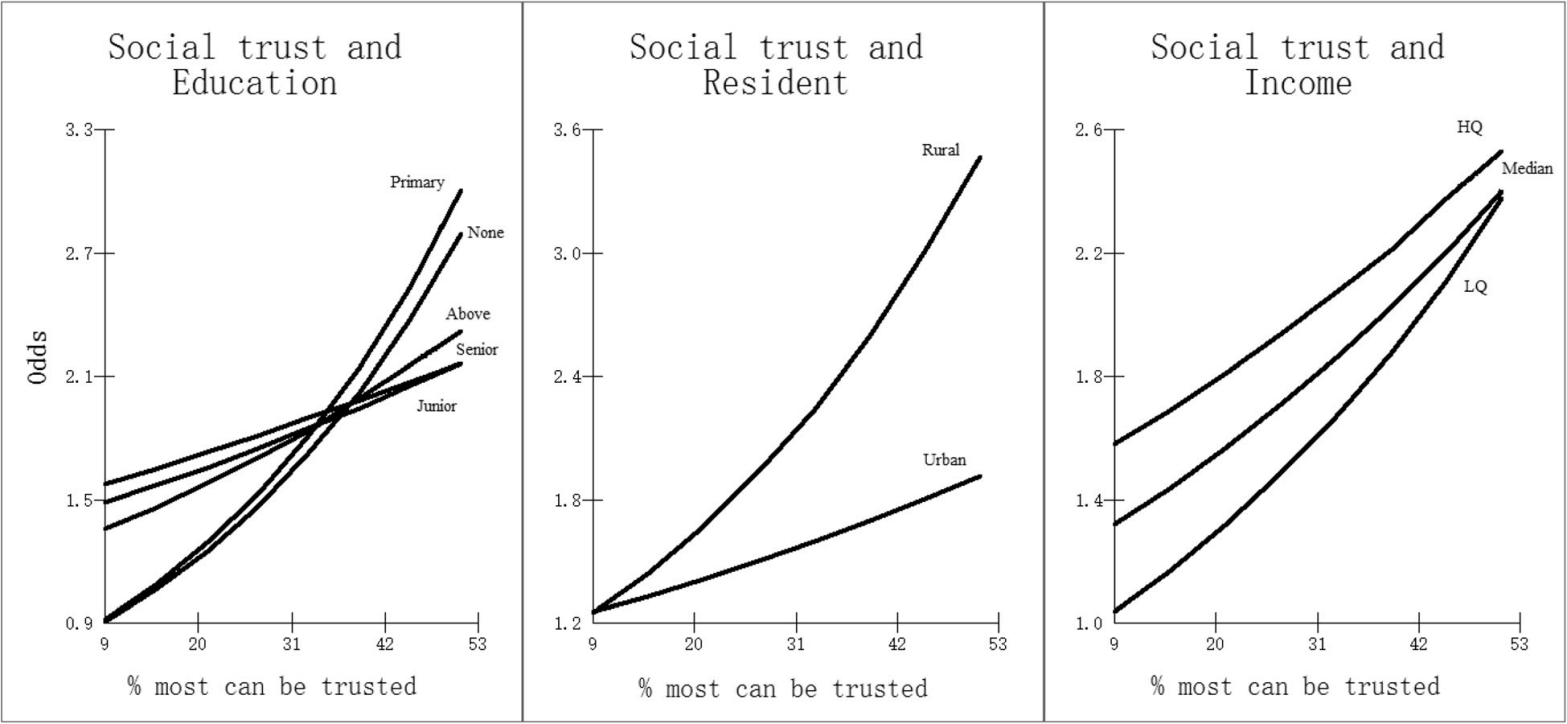
Cross-level interactions with social trust (derived from Model 4A_2005, 4B_2005 and 4C_2005) (odds of reporting good health) (“Out-group” social trust)

Model 4 shows the further estimation for cross-level interactions between each individual predictor and the “Out-group” social trust variable (province level). The interaction between social trust and educational attainment (Model 4A_2005), the interaction between social trust and rural/urban residence (Model 4B_2005) and the interaction between social trust and income (Model 4C_2005) show a lower DIC than Model 3B, which suggests an improvement of model fit. The results of these three multi-level logit models are also shown in Table 2. For ease of interpretation for each of the cross-level interactions, the logits were transformed to odds ratios (ORs) in Fig. 1 using the simulation-based procedures of the *MLwiN* Customised predictions facility [10, 19]. In terms of educational attainment, the odds of reporting good health for all the education groups increase with increasing “out-group” social trust. Individuals with primary levels of education or no formal education are the least likely to report good health than the other educational attainment groups in provinces with low “out-group” social trust; while, such individuals are much more likely to report good health than the other educational attainment groups in provinces with high “out-group” social trust. Divergent trends are found for rural and urban residents. There is little difference between rural and urban residents’ report of good health in provinces with low “out-group” social trust, however this gap widens with higher “out-group” social trust and the increase is most marked for rural residents. In contrast, convergent trends are found for the different income groups, represented by the Lower Quartile (LQ = 25 %), the Median (MQ = 50 %) and Higher Quartile (HQ = 75 %). Individuals in the Lower Quartile are the least likely to report good health in a low “out-group” social trust province; while, the gaps between the income groups narrow with increasing “out-group” social trust.

In terms of the results of “in-group” trust and health in 2010, the model strategy remained the same as for the “out-group” trust model (Models 1 to 3B). Tables 3 and 4 presents the results of the multi-level logit models for “in-group” trust and health in 2010. In contrast to a positive effect of “out-group” social trust on health in 2005, high “in-group” social trust now shows a negative association with the report of good health (ORs = 0.97, 95 % CI: 0.95-1.00); while, individuals who reported that most people can be trusted are 42 % more likely to report good health than those who think that most people cannot be trusted (95 % CI: 1.27-1.58). For the control variables, similarly to the results of the 2005 data, older people, women, those with no formal educational attainment and those in low income groups in general report worse health than younger people, men, those with higher educational attainments (junior or senior level), and those on higher incomes respectively. There are no significant differences in self-rated health found between urban and rural residents.

**Table 3:** Logit multi-level regression estimates for 2010 (“In-group” trust)

	Model 1	Model 2	Model 3A	Model 3B
	Estimate (S.E.)	Estimate (S.E.)	Estimate (S.E.)	Estimate (S.E.)
Fixed Part				
CONS	0.106 (0.061)**	-0.159 (0.116)	-0.209 (0.138)	-1.390 (1.049)
Age		-0.043 (0.002)***	-0.045 (0.002)***	-0.045 (0.002)***
Male (ref: Female)		0.225 (0.046)***	0.231 (0.047)***	0.230 (0.049)***
Educational attainment (ref: None)				
Primary		0.247 (0.078)***	0.246 (0.079)***	0.243 (0.080)***
Junior		0.324 (0.083)***	0.325 (0.085)***	0.323 (0.085)***
Senior		0.429 (0.089)***	0.424 (0.093)***	0.423 (0.091)***
Above		0.544 (0.110)***	0.519 (0.113)***	0.513 (0.111)***
Rural (ref: urban)		0.034 (0.055)	0.007 (0.057)	0.013 (0.054)
Log personal income		0.173 (0.023)***	0.170 (0.023)***	0.169 (0.023)***
Log personal income^2^		0.025 (0.011)**	0.025 (0.011)**	0.024 (0.011)**
Interpersonal trust (ref: Low trust)				
Average trust			0.036 (0.083)	0.037 (0.084)
High trust			0.345 (0.053)***	0.346 (0.055)***
Social trust				-0.028 (0.014)**
*Interactions:*				
Social trust * Age				
Social trust * Education (ref: Social trust * None)				
Social trust * Primary				
Social trust * Junior				
Social trust * Senior				
Social trust * Above				
Social trust * Log personal income				
Social trust * Interpersonal trust (ref: Social trust * Low trust)				
Social trust * Average trust				
Social trust * High trust				
Random Part				
Level 2				
Between Province	0.204 (0.062)***	0.230 (0.070)***	0.240 (0.074)***	0.211 (0.066)***

***p < 0.01, **p < 0.05, *p < 0.1

^2^ stands for the Square form of log personal income in the model

**Table 4:** Logit multi-level regression estimates for 2010

	Model 4A_2010	Model 4B_2010	Model 4C_2010	Model 4D_2010
	Estimate (S.E.)	Estimate (S.E.)	Estimate (S.E.)	Estimate (S.E.)
Fixed Part				
CONS	-0.288 (0.093)***	-0.284 (0.097)***	-0.259 (0.087)***	-0.320 (0.089)***
Age	-0.045 (0.002)***	-0.045 (0.002)***	-0.045 (0.002)***	-0.045 (0.002)***
Male (ref: Female)	0.230 (0.046)***	0.231 (0.047)***	0.227 (0.047)***	0.234 (0.047)***
Educational attainment (ref: None)				
Primary	0.241 (0.078)***	0.227 (0.079)***	0.241 (0.078)***	0.244 (0.081)***
Junior	0.321 (0.083)***	0.308 (0.085)***	0.311 (0.084)***	0.321 (0.083)***
Senior	0.423 (0.093)***	0.407 (0.095)***	0.413 (0.092)***	0.420 (0.092)***
Above	0.519 (0.112)***	0.524 (0.116)***	0.511 (0.111)***	0.510 (0.113)***
Rural (ref: urban)	0.014 (0.056)	0.013 (0.056)	0.019 (0.055)	0.016 (0.054)
Log personal income	0.170 (0.022)***	0.170 (0.023)***	0.177 (0.023)***	0.170 (0.023)***
Log personal income^2^	0.026 (0.010)**	0.025 (0.010)**	0.028 (0.010)**	0.025 (0.010)**
Interpersonal trust (ref: Low trust)				
Average trust	0.037 (0.085)	0.030 (0.081)	0.038 (0.084)	0.027 (0.083)
High trust	0.342 (0.053)***	0.344 (0.054)***	0.348 (0.053)***	0.336 (0.054)***
Social trust	-0.029 (0.015)**	-0.063 (0.020)***	-0.029 (0.014)**	-0.012 (0.018)
*Interactions:*				
Social trust * Age	-0.001 (0.000)***			
Social trust * Education (ref: Social trust * None)				
Social trust * Primary		0.033 (0.015)**		
Social trust * Junior		0.037 (0.015)**		
Social trust * Senior		0.031 (0.017)*		
Social trust * Above		0.052 (0.020)**		
Social trust * Log personal income			0.014 (0.004)***	
Social trust * Interpersonal trust (ref: Social trust * Low trust)				
Social trust * Average trust				0.012 (0.018)
Social trust * High trust				-0.038 (0.011)***
Random Part				
Level 2				
Between Province	0.210 (0.064)***	0.209 (0.064)***	0.217 (0.070)***	0.216 (0.066)***

***p < 0.01, **p < 0.05, *p < 0.1

^2^ stands for the Square form of log personal income in the model

In terms of the cross-level interactions of “in-group” social trust (province level) and each individual predictor, only the interaction between “in-group” social trust and age (Model 4A_2010), the interaction between “in-group” social trust and educational attainment (Model 4B_2010), the interaction between “in-group” social trust and income (Model 4C_2010), and the interaction between “in-group” social trust and “in-group” interpersonal trust (Model 4D_2010) show a lower DIC than in Model 3B. The results of multi-level logit models are also shown in Tables 3 and 4.

**Fig. 2:**
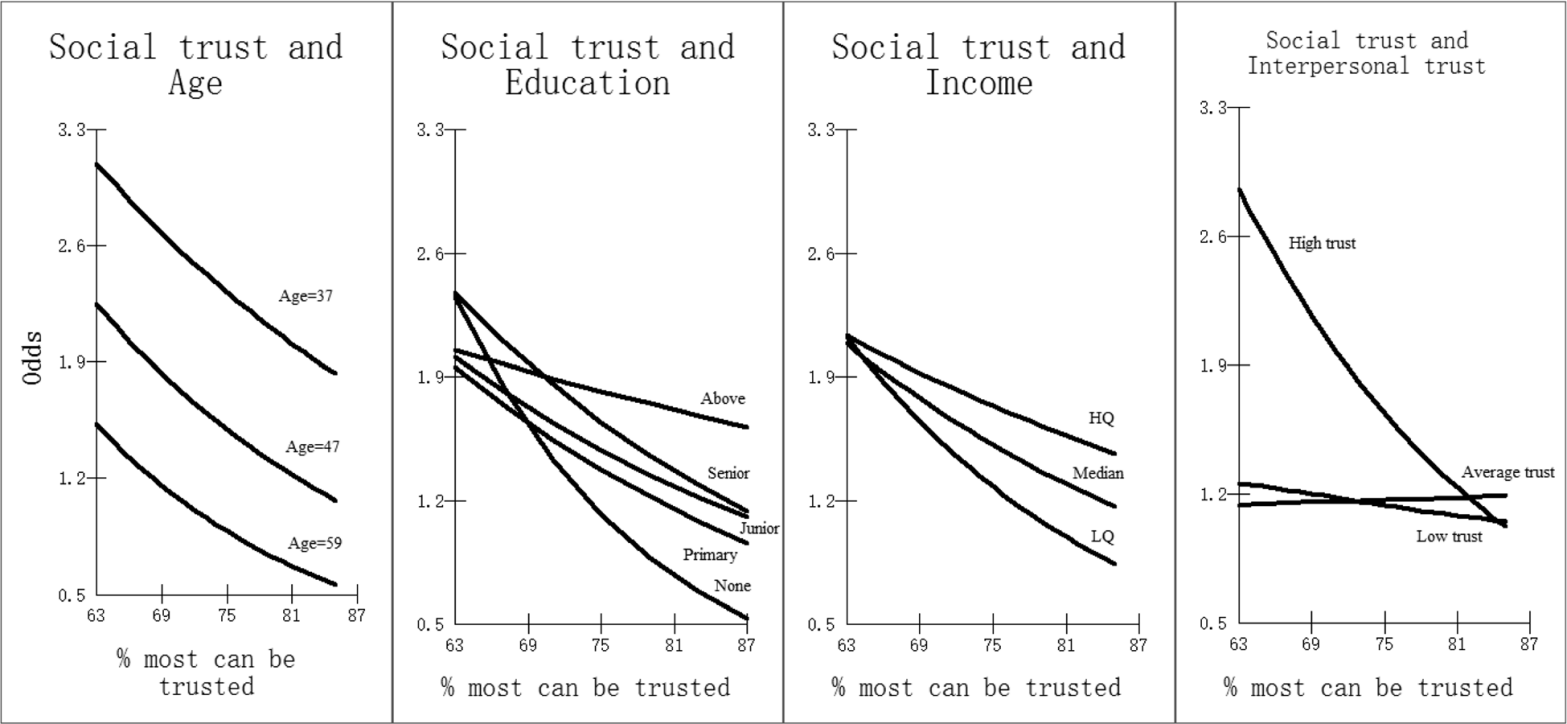
Cross-level interactions with social trust (derived from Model 4A_2010, 4B_2010, 4C_2010 and 4D_2010) (odds of reporting good health) (“In-group” social trust)

The odds for cross-level interactions from models 4A_2010, 4B_2010, 4C_2010 and 4D_2010 are presented in Fig. 2. The age effects are plotted for respondents at three age groups: 37-46 (Lower quartile), 47-58 (Median) and 59 and over (Upper quartile). At each age bracket, there are decreased odds of reporting good health as the percentage of “in-group” social trust (at the province level) increases. Divergent trends are found for different levels of educational attainment. There is little difference among individuals with different levels of educational attainment reporting good health with low “in-group” social trust, however this gap widens with higher social trust, and the decrease is most marked for those without any formal education. In terms of income, all of the income quartile groups are less likely to report good health with an increasing percentage of “in-group” social trust. Divergent trends are found for these three groups’ report of good health in provinces with low “in-group” social trust. However, this gap grows with increasing “in-group” social trust and those in the Lower Quartile income bracket are much less likely to report good health in high “in-group” social trust provinces. “In-group” social trust at the province level has a substantial influence for individuals who think that most people can be trusted. The odds of reporting good health decrease dramatically for those who think that most people can be trusted from a province with low “in-group” social trust to a province with high “in-group” social trust. No substantial change in self-rated health is found between the low and high “in-group” social trust environments for those with average or low trust.

## Discussion

The aim of this study was to investigate the relationship between trust and health in China. Since China has a different culture and measurements of the concepts of trust from Western countries, we paid particular attention to two concepts of trust (namely “out-group” trust and “in-group” trust) and the effects of these concepts at both the individual and societal levels on people’s health in China. The main findings show that higher “out-group” social trust at the societal level (province) is associated with the report of good health; while, higher “in-group” social trust at the societal level is negatively associated with reporting good health. Although Chinese communities are characterized as “low-trust” societies in terms of “out-group” trust, the findings in the 2005 data are consistent with previous studies (i.e. [24]): namely, trust has protective effects for health [17]. Beyond concrete personal connections, Chinese individuals have a “pervasive distrust of strangers” [3] which hinders the cooperation between people, solidarity, spontaneous sociability, a tolerant society, a vibrant social community, and health-related behaviour [4]. Such distrust could reduce the social quality of people’s lives, thus depressing the likelihood of reporting good health in a society with low “out-group” trust; however, from the results in 2010, it was found that societies with higher “in-group” trust do not show better health outcomes.

Such a finding may be due to the Chinese culture and particularly the concept of nepotism. Chinese culture has long been a prototype of a familist and trust-discouraging tradition and it is characterized by a narrow “radius of trust” (from core family members to less familiar persons) ([3, 23], pp.69-95). Higher “in-group” trust societies in China may reflect closer personal connections (networks), where individuals trust their own networks’ members (i.e. family member, relative, friend, colleague and people from the same hometown); conversely, people outside such concrete relationships are hard to be trusted, and the sense of not being considered trustworthy may lead to chronic stress in one’s daily life and may thus increase the risk to one’s health [13]. In addition, moral behaviour in the Chinese culture is closely linked to personal connections [23] and the display of trust is linked more to creating “impressions” towards other individuals rather than making rational judgments [14]. A high “in group” trust society may also reflect a society based on acquaintances which emphasises the “rule of man” rather than the “rule of law”. In this context, the working ethic is primarily based on the degree of closeness of relationships; individuals are more likely to be chosen by core members to achieve their goals if they have a closer and more familiar relationship. Consequently, for those people who are not members of one’s “in-group”, their rights may be violated due to unfair judgments which come as a result of a lack of transparency in rights and responsibilities in terms of public affairs [21]. Therefore, it could be argued that high “in-group” trust societies are not conducive to individuals’ good health. In terms of interpersonal trust, our analyses find that individuals who consider that strangers or most people can be trusted are more likely to report good health than those who consider strangers/most people as untrustworthy.

In addition, some particular groups are more strongly impacted by social trust at the province level than other groups according to the cross-level interaction results. In terms of “out-group” social trust, our findings show that the odds of reporting good health for those with lower levels of educational attainment increase more rapidly than for those with high levels of educational attainment in high “out-group” social trust provinces. Education is stable throughout life and helps people to think logically and rationally, and to analyse problems and solve them; it could also help to solve interpersonal problems in terms of social interaction through shaping people’s communication skills and cognitive flexibility (i.e. negotiating with each other, learning to compromise, respond flexibly and openly to others’ opinions rather resorting to violence) [20]. On the other hand, education has also been regarded as the most effective way of internalizing social norm in traditional Confucianism culture which is prevalent in China, and individuals with higher education are treated as more trustworthy than those with lower education [14].

Three pathways could explain how education can affect trust in China: people’s personality can be shaped by education through embedding honesty into one’s rational behaviour so as to be well received by others in social transactions; higher educational attainment not only can enhance one’s human capital which could improve one’s earnings and social status, but also can enhance trustworthiness; and education could accelerate people’s social participation which conversely facilitates information flow between members of society [14]. Individuals with lower education may not only be less likely to be considered trustworthy, but they may also be more sensitive to such a disadvantage and therefore more prone to assimilate in a social trust environment than well-educated people. Therefore, in higher “out-group” trust societies, lower education attainment may lead to a stronger feeling of being trustworthy which in turn could result in better health. By contrast, in higher “in-group” trust societies, low-educated individuals may find it more difficult to be trustworthy and thus be affected more negatively by a culture of nepotism, which could in turn result in a lower likelihood of reporting good health.

In terms of the differences between rural and urban residents, the traditional beliefs and social structures in rural areas involve less radical ideological transformations than in urban areas; the social networks in rural areas are more traditional and “tighter knit” than those in urban areas, and interaction with strangers is much more restricted for rural residents in China [23]. Therefore, higher “out-group” trust societies could result in rural residents having stronger feelings of being trustworthy than urban residents which could accelerate better health for rural residents compared to their urban counterparts. Apart from the reduced likelihood of being able to afford health care consumption and medical expenditures, individuals on lower incomes are often marginalized and the social distance between them and those who are “well-off” is extended in an unequal society [18]. Additionally, the sense of not being trustworthy, as well as the feeling of shame and exclusion, may lead to chronic stress in one’s daily life thus increasing the risk of poor health [13]. This could be the reason why the gaps between individuals on lower and higher income are smaller in high “out-group” social trust areas than in low “out-group” social trust areas.

In terms of “in-group” social trust at the province level, the explanation for the different levels of income groups in social trust is similar as previously stated. The feeling of exclusion from networks of well-off people or being isolated from other individuals’ personal connections may be more harmful for the health of those on lower incomes in high “in-group” social trust areas. For the cross-level interaction between “in-group” interpersonal trust (individual level) and “in-group” social trust (societal level), the odds of reporting good health dramatically decrease between provinces with low “in-group” social trust and provinces with high “in-group” social trust for individuals who think that most people can be trusted (high trust). Trust is a two-way process, therefore if high trust individuals know that they are not trustworthy or they are not able to be ones’ “in-group” members and be trusted by others in their personal networks, they may feel disappointment and shame, thus increasing the risk to their health. By contrast, individuals who think that most people cannot be trusted or “as many can be trusted as distrusted”, are already less likely to trust others, and it seems that they have lower expectations of being trustworthy [6], therefore, whether they live in high or low “in-group” social trust areas, the effect of distrust on health remains the same for them.

Three limitations in this study were recognized. The CGSS does not provide a consistent questionnaire on “out-group” trust and “in-group” trust between 2005 and 2010. In addition to shedding light on this relationship in a non-western society, this study also fills gaps in contemporary knowledge of “in-group” / “out-group” trust and health in the context of China. The second limitation of the study is its repeated cross-sectional nature, which does not allow us to draw conclusions on the causal direction of the relationship between trust and health status. Although the examination of such relationship with longitudinal data would be beneficial, nevertheless from our knowledge, there is currently no available longitudinal survey which has such consistent information about trust and health in China. The third limitation could the geography layer. Measurement of social trust in the province level may not appropriate; however, the changed survey methods of CGSS from county level in 2005 to community level in 2010 could result in inconsistent measure scale of social trust. On the other hand, the population weight the CGSS provided is based on province level, the weighted social trust in province level could reflect the real social trust environment in China. Despite these limitations, the findings in this paper fill the gap in our knowledge of trust and health in the context of China and provide additional insight into the effects of “in-group” trust and “out-group” trust on health in China.

Confucian social ethics have been the dominant traditional culture in China, and such ethics are also emphasized by the Chinese government [23]. Although there is a Confucian emphasis on ritual and civility in concrete personal relationships, this lack of “rules for impersonal dealings beyond the face-to-face level” also means that there is a lack of trust that extends beyond the realm of concrete relationships [23]. Other research has noted that trust in China is based more upon “impression” rather than rational judgment [14]. A key challenge to policy implementation is how to improve the “out group” social trust thereby improving the population health. First, Torpe and Lolle [27] found that high educational attainment was positively associated with high social trust (“out group”) in western countries; therefore, governments could invest more on educational attainment across the population. Higher levels of educational attainment not only could improve social trust, but also could protect people’s health even in a society with low “out-group” trust. Education could also help to improve people’s rational judgments in daily life which could improve interpersonal communication. Second, income inequality between the top and bottom levels of society might be an obstacle for the development social trust. Effective social security (i.e. health care, health insurance, unemployment insurance), may help to reduce the inequalities and thus improve the social trust and health outcomes. Finally, the establishment of rules for impersonal dealings beyond the face-to-face levels, which can improve personal connections with strangers beyond individuals’ personal networks, could also encourage individuals to develop more trust within society.

## Conclusion

This paper found that higher levels of interpersonal trust are associated with good health. At the province level, higher “out-group” social trust is positively associated with better health; while, higher “in-group” social trust is negatively associated with good health. Individuals with lower levels of educational attainment (no education or only primary level), rural residents and those on lower incomes were the most affected groups compared with their counterparts in higher “out-group” trust society; while, individuals with lower levels of educational attainment (no education or only primary level), those who think that most people can be trusted in interpersonal and those on lower incomes were the most affected groups compared with their counterparts in higher “in-group” trust society.

## Abbreviations

CGSS: China General Social Survey
CI: Confidence Interval
HQ: Higher Quartile
LQ: Lower Quartile
MQ: Median Quartile
ORs: Odds Ratios
SRH: Self-Rated Health

## Footnotes

1 All of these studies have controlled for key demographic and socioeconomic characteristics (i.e. age, gender, educational attainment, and income).

2 In 2005, the CGSS surveyed 28 of the 31 provinces in mainland China, Tibet, Qinghai and Ningxia are not included in these datasets; in 2010, the CGSS surveyed all 31 provinces in mainland China.
